# Health Care Professionals’ Experiences With a Mobile Self-Care Solution for Low Complex Orthopedic Injuries: Mixed Methods Study

**DOI:** 10.2196/51510

**Published:** 2024-02-02

**Authors:** Jelle Spierings, Gijs Willinge, Marike Kokke, Bas Twigt, Wendela de Lange, Thijs Geerdink, Detlef van der Velde, Sjoerd Repping, Carel Goslings

**Affiliations:** 1 Department of Traumasurgery St Antonius Hospital Utrecht Netherlands; 2 Onze Lieve Vrouwe Gasthuis The Department of Trauma Surgery Amsterdam Netherlands; 3 The Healthcare Innovation Center Julius Center for Health Sciences and Primary Care University Medical Center Utrecht Utrecht Netherlands; 4 Department of Public Health Amsterdam UMC Amsterdam Netherlands

**Keywords:** application, direct discharge, eHealth, experience, healthcare professional, mixed method study, orthopaedic surgery, orthopaedic, policy, policymaker, self-care application, self-care, trauma surgery, utilization, virtual fracture clinic

## Abstract

**Background:**

To cope with the rising number of patients with trauma in an already constrained Dutch health care system, Direct Discharge (DD) has been introduced in over 25 hospitals in the Netherlands since 2019. With DD, no routine follow-up appointments are scheduled after the emergency department (ED) visit, and patients are supported through information leaflets, a smartphone app, and a telephone helpline. DD reduces secondary health care use, with comparable patient satisfaction and primary health care use. Currently, little is known about the experiences of in-hospital health care professionals with DD.

**Objective:**

The aim of this study was to explore the experiences of health care professionals with the DD protocol to enhance durable adoption and improve the protocol.

**Methods:**

We conducted a mixed methods study parallel to the implementation of DD in 3 hospitals. Data were collected through a preimplementation survey, a postimplementation survey, and semistructured interviews. Quantitative data were reported descriptively, and qualitative data were reported using thematic analysis. Outcomes included the Bowen feasibility parameters: implementation, acceptability, preliminary efficacy, demand, and applicability. Preimplementation expectations were compared with postimplementation experiences. Health care professionals involved in the daily clinical care of patients with low-complex, stable injuries were eligible for this study.

**Results:**

Of the 217 eligible health care professionals, 128 started the primary survey, 37 completed both surveys (response rate of 17%), and 15 participated in semistructured interviews. Health care professionals expressed satisfaction with the DD protocol (median 7.8, IQR 6.8-8.9) on a 10-point scale, with 82% (30/37) of participants noting improved information quality and uniformity and 73% (27/37) of patients perceiving reduced outpatient follow-up and imaging. DD was perceived as safe by 79% (28/37) of participants in its current form, but a feedback system to reassure health care professionals that patients had recovered adequately was suggested to improve DD. The introduction of DD had varying effects on workload and job satisfaction among different occupations. Health care professionals expressed intentions to continue using DD due to increased efficiency, patient empowerment, and self-management.

**Conclusions:**

Health care professionals perceive DD as an acceptable, applicable, safe, and efficacious alternative to traditional treatment. A numerical in-app feedback system (eg, in-app communication tools or recovery scores) could alleviate health care professionals’ concerns about adequate recovery and further improve DD protocols. DD can reduce health care use, which is important in times of constrained resources. Nonetheless, both advantages and disadvantages should be considered while evaluating this type of treatment. In the future, clinicians and policy makers can use these insights to further optimize and implement DD in clinical practice and guidelines.

## Introduction

The global increase in the number of patients with trauma presents a major challenge to the already strained health care systems [1,2]. To achieve more sustainable health care, digital alternatives to face-to-face outpatient follow-up have been introduced as a supported strategy [3-5]. These alternatives, also known as “eHealth,” are defined by Eysenbach [6] as the intersection of medical informatics, public health, and business, referring to health services and information delivered or enhanced through the internet and related technologies. In a broader sense, the term characterizes not only a technical development but also a state of mind; a way of thinking; an attitude; and a commitment to networked, global thinking to improve health care locally, regionally, and worldwide by using information and communication technology.

Based on a British example, a Dutch teaching hospital implemented the Direct Discharge (DD) protocol to maintain the quality of care for patients with trauma in 2019 [7,8]. The DD protocol involves discharging patients from the emergency department (ED) without scheduled follow-up while providing patients with information through a self-care mobile eHealth app. DD significantly reduces secondary health care use (SHU) with similar levels of patient satisfaction and primary health care use (PHU) [9-11]. Based on these results and catalyzed by the COVID-19 pandemic, over 25 Dutch hospitals have implemented this protocol since 2019 [12].

The successful and sustainable adoption of digital health technology is complex and influenced by various factors at organizational, technological, and social levels [13,14]. This complexity is widely recognized in eHealth and eHealth evaluation frameworks [15-17]. The organizational and logistic benefits and patient satisfaction scores following DD have been well described in the literature [18-20]. However, the social aspects of the DD protocol for health care professionals remain underreported [21]. An in-depth exploration is warranted to better understand the adoption of the DD protocol within its social context, including insight into the experiences of stakeholders. The aim of this study was to explore the experiences of health care professionals with the DD protocol parallel to the implementation of this protocol in 3 Dutch hospitals to enhance durable adoption and improve the protocol.

## Methods

### Design

An observational mixed methods study was conducted among health care professionals from August 2021 to June 2022, parallel to the implementation of DD in 3 hospitals. Both quantitative and qualitative data were collected and analyzed separately by a quantitative team (GW and JS) and a qualitative team (WDL and Elke Mathijssen). The Bowen feasibility framework was used to organize both data sources with the following parameters: implementation, acceptability, preliminary efficacy, demand, and applicability [22]. After separate analyses, quantitative and qualitative data were triangulated with the Pillar Integration Process [23]. This study was reported according to the Good Reporting on a Mixed Methods Study (GRAMMS) criteria (Multimedia Appendix 1) [24].

### Context

**Figure 1:**
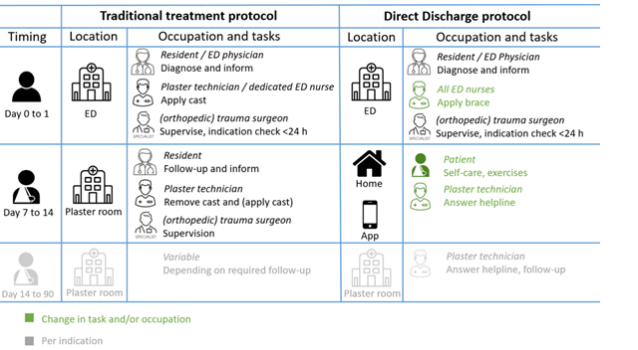
Treatment protocols before and after implementation of DD and changes in location, involved stakeholders and tasks. DD: Direct Discharge; ED: emergency department.

The 3 participating centers were urban, level-2 trauma centers with up to 3 locations per hospital, treating between 1200 and 1800 patients with low-complex traumatic musculoskeletal injuries annually. Each center had a similar size and structure. All centers consisted of 3 locations, with 1 large location focusing on low-to-high complex traumatic injuries and having an ED with more rooms compared with the other locations. The 2 other locations were smaller and had no particular focus on patients with trauma, but they treated low-complex patients with trauma if they sought care at these locations. Per center, all 3 locations have 1 team taking care of all patients. These teams consisted of (orthopedic) trauma surgeons, residents, plaster technicians, ED physicians, and ED nurses. In total, 217 eligible health care professionals were exposed to DD based on data provided by participating hospitals. Changes in tasks per health care professional are described in Figure 1. These changes apply per center, including all 3 hospitals per center. The variance in the number of employees was correlated with the size of the hospital. The 2 centers implemented DD in September 2021 and 1 in March 2022.

### Traditional Treatment

Before DD was implemented, patients were treated according to the local trauma protocols. These protocols consisted of immobilization or support with either a cast, sling, bandage, or splint and brief information about the injury at the ED. At least 1 outpatient follow-up appointment was scheduled at the plaster room or in the outpatient clinic within 2 weeks after the injury for review, extensive information, and definitive management planning.

### Direct Discharge Protocol

This protocol was derived from the British model of a Virtual Fracture Clinic (VFC) and adapted to the Dutch health care setting in 2019 [25]. In its Dutch adaptation, DD includes 11 treatment protocols for low-complex, stable traumatic orthopedic injuries with additional injury-related criteria (Multimedia Appendix 2) [25]. If patients met the injury-related inclusion criteria in Multimedia Appendix 2 and spoke Dutch or English fluently, they were included; no further predefined restrictions (eg, age or comorbidity) were used. Patients were excluded from the protocol at the ED if they had initial treatment in another hospital; follow-up in another hospital (eg, closer to home); multiple injuries; a reason for follow-up other than the injury (eg, social-care reasons); an eye-, motor-, or verbal-score <15 at presentation; or intoxication. With DD, patients were discharged directly from the ED without routine outpatient follow-up. They received a removable orthosis or a sling (eg, brace instead of a cast) and extensive information at the ED, summarized in a mobile self-care app (the VFC app). Patient eligibility for the protocol was re-evaluated on the next workday (within 24 hours) by a team consisting of an (orthopedic) trauma surgeon and a radiologist. If patients were incorrectly discharged directly based on the injury-related or social inclusion criteria during the second review the next day, then they were contacted by phone and scheduled for a face-to-face appointment. This re-evaluation was a standard procedure in both protocols and was used to check previous decisions of young doctors by a senior group of medical professionals based on the radiographs and electronic patient record.

### The VFC App

**Figure 2:**
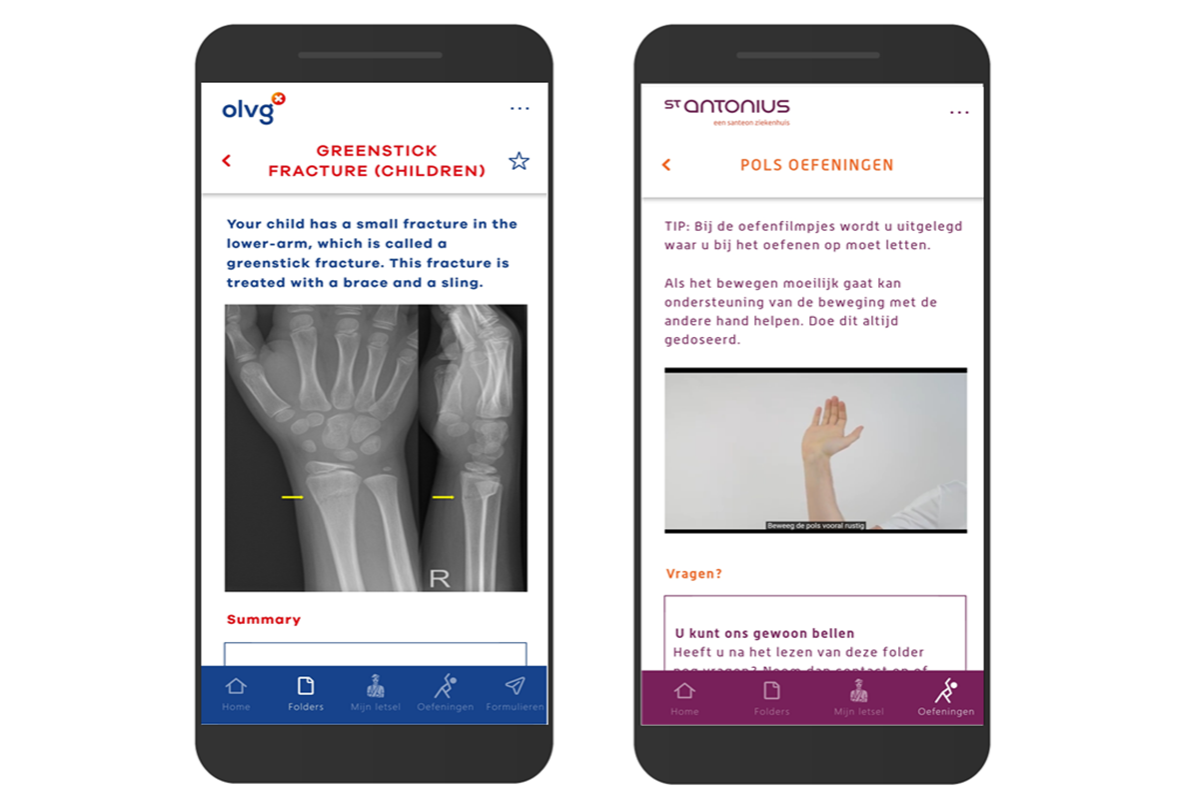
English and Dutch in-app screenshots of the Virtual Fracture Clinic app used in the Direct Discharge protocol.

The VFC app provides self-care assistance through information, videos, and a helpline and can be downloaded for free at the Google Play Store and iOS App Store (Figure 2). Injury-specific leaflets with recovery information, treatment rules, and red flags were included. Furthermore, frequently asked questions, audiovisual exercise, immobilization, and analgesic instructions were included to assist patients. If patients required human contact in addition to the information, a helpline by phone operated by a health care professional was available during working hours. The VFC app aimed to increase self-management and self-care during recovery and to substitute face-to-face follow-up.

### Implementation Tools and Materials

Interested centers contacted 1 of the trauma surgeons or the main email address of the initiating Onze Lieve Vrouwe Gasthuis (OLVG) Hospital, Amsterdam, the Netherlands. The OLVG Hospital created several tools to assist the implementation in other hospitals. The tools were created to streamline the implementation process as hospitals had similar questions during implementation and medical doctors struggled with the implementation of eHealth in their daily practice, partly due to inexperience with implementation and accompanying barriers and facilitators. These tools consisted of an email address that health care professionals could contact, an implementation guide, a digital PowerPoint presentation with an overview of the concept, and an information set with standardized information. This information set included posters, pocket cards, training guides, smart phrases for electronic patient records, standardized discharge letters, and a tool to personalize the layout of the VFC app. Participating centers started implementation preparations 3 months before the actual start of DD as the standard of care. Based on the experiences of the implementation in previous centers, 3 months was considered a sufficient amount of time to implement DD. There were minor differences between hospitals to optimize local fit (eg, the available hours of the staffed helpline).

### Study Population

Health care professionals involved in the in-hospital treatment of patients with eligible low-complex, stable traumatic orthopedic injuries on a daily basis were included in this study (Figure 1). Health care professionals were excluded from the final analysis if they did not provide the correct contact details or did not complete both surveys.

### Sampling and Recruitment

**Figure 3:**
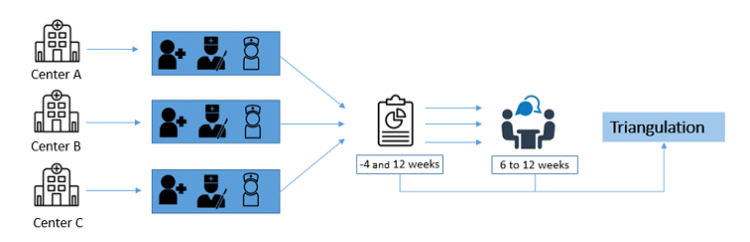
Summary of hospitals, procedures, and models used to evaluate the Direct Discharge protocol among health care professionals. All centers consisted of 3 hospitals with similar teams and similar sizes.

A total of 4 weeks before the implementation of DD, the 217 potentially eligible health care professionals were asked to participate in the study through education moments, e-learnings, and by email. The health care professionals were given a survey distribution link by email to a preimplementation survey in Research Electronic Data Capture (REDCap; Vanderbilt University), a digital survey system [26]. Potentially eligible participants were remembered twice, 1 week after the initial email. Participants were excluded if they did not complete both surveys or if they did not provide any contact details to send the second survey to. Within the survey, consent for an additional semistructured interview was asked. Age, sex, occupation, medical specialty, and hospital were used to select a purposive sample among health care professionals who consented. During sample selection for the interviews, ED nurses and plaster technicians were underrepresented. Through an open call, nonresponders were recruited through email, after which the definitive sample was selected. Eligible health care professionals were contacted to schedule a web-based, semistructured interview. Health care professionals were reminded through email to complete the survey. The second survey was sent 3 months after the implementation of the DD protocol (Figure 3). We aimed to collect completed quantitative data from 100 health care professionals and qualitative data from 15 health care professionals.

### Data Collection

Data were collected from surveys and semistructured interviews.

#### Surveys

A total of 2 surveys, a preimplementation survey and a postimplementation survey (Multimedia Appendix 3), with 46 questions, including close-ended questions, multiple-choice questions, 5-point Likert scales, visual analogue scales, and free-text questions, measured 5 Bowen feasibility parameters. As no golden standard for the evaluation of innovations exists, the surveys and topic list were developed by 4 researchers (JS, GW, BT, and TG) and checked by 2 experts on relevance: a professor in trauma surgery (CG) and an associate professor in process evaluations of health care innovations (Jaap Trappenburg). We pretested the survey with 5 health care professionals to improve clarity. After data collection, the preimplementation expectations were compared with the postimplementation experiences.

#### Semistructured Interviews

A total of 2 independent researchers from “The Healthcare Innovation Centre,” not involved in daily clinical practice or the VFC research team, conducted digital semistructured interviews to minimize social-desirability bias. The web-based interviews were held at least 8-12 weeks after the implementation of DD to warrant an optimal recall. The interviews were guided by a topic list based on literature, including the previously mentioned Bowen feasibility parameters (Multimedia Appendix 4). The research team piloted the topic list for clarity and completeness and modified it during data collection. A pragmatic choice was made to interview 5 different types of health care professionals from each participating center, which was deemed sufficient to get a good understanding of the experiences of health care professionals and to reach saturation. No new themes were identified in the last step of the analysis, indicating that saturation was reached.

### Data Analysis

#### Quantitative Data

Quantitative data were analyzed using SPSS (version 27; IBM Corporation) [27]. Baseline characteristics and outcomes were reported descriptively using numbers and proportions for categorical variables and mean (SD) or median (IQR) for continuous variables as appropriate. The normal distribution of continuous data was assessed with a visual analysis of boxplots. The paired 2-tailed *t* test or Mann-Whitney *U* test was used to determine the statistical significance of parametric variables for normally and nonnormally distributed data, respectively.

#### Qualitative Data

Qualitative data were analyzed using an inductive approach. Data analysis started after the first 5 interviews. Interviews were audiotaped, summarized, and analyzed using NVivo (version 12; QSR International) [28]. A total of 2 researchers (WDL and Elke Mathijssen) used inductive analysis with methods to ensure reliability and validity [29,30]. The data was independently analyzed by 1 researcher (WDL), and another researcher (Elke Mathijssen) reviewed the analysis. Discrepancies and remarks were discussed until they reached a consensus about the interpretations of the data. Memos were made to track research decisions during analysis. Code saturation was reached when no new categories or themes emerged from the new raw data [31]. We considered 15 interviews sufficient to reach saturation and get a good understanding of the experiences of professionals. Therefore, the number of interviews was limited by a pragmatic choice of available time. The final themes were used to describe the value and feasibility of DD from the perspective of involved health care professionals.

#### Triangulation

After the separate quantitative and qualitative analyses, the findings were triangulated with a simplified approach of the Pillar Integration Process technique [23]. This approach uses a transparent and rigorous 4-stage technique for integrating and presenting qualitative and quantitative findings in a joint display (Microsoft Excel, 2018; Microsoft Corporation) [32]. A researcher (JS) presented the quantitative findings per study parameter, and another presented the qualitative findings (WDL). Dissimilarities and self-contained themes were objectified. These themes were merged by 1 of the researchers (Elke Mathijssen) into a meaningful narrative (the pillar), which was reviewed by 2 researchers (JS and WDL).

### Patient and Public Involvement

Patients or health care professionals were not involved in the design, intervention, research question, or outcome measures of this study.

### Ethical Considerations

This study, including the process analysis, was reviewed and approved by the Medical Ethical Committee of Utrecht, Netherlands (W21.261).

Patients provided consent for participation in the research, and could opt out at any time after request by e-mail. The original consent or IRB approval covers secondary analysis without additional consent. Data is de-identified. A data key is stored at the local hospitals in a secured map and coded file. This is only accessible to JS and GW. The accessible data has been de-identified as far as possible (e.g., age in years instead of date of birth). Patients received no compensation to participate in this research.

## Results

### Demographics

**Figure 4:**
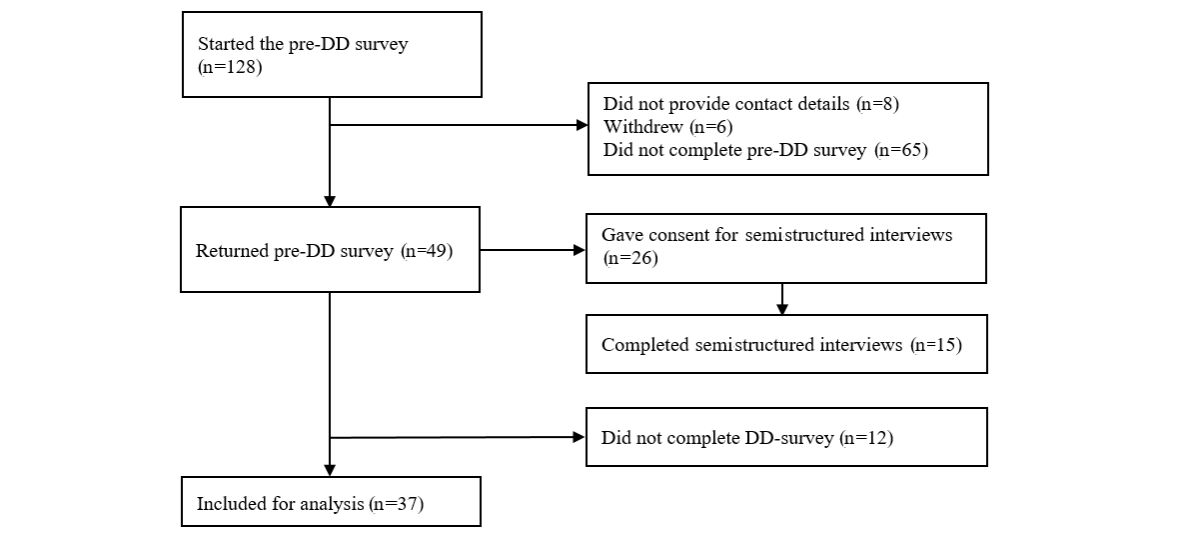
Flow diagram of the included health care professionals in the evaluation of the Direct Discharge (DD) protocol.

Of the 217 estimated eligible health care professionals, 128 started the primary survey, 42 did not complete the primary survey, and 49 did not complete both surveys (Figure 4). Of the 37 included health care professionals (response rate of 17%), 23 (62%) were female, and the median age was 38 (IQR 32-45) years. Current occupations were medical specialists (14/37, 38%), residents (14/37, 38%), plaster technicians (7/37, 19%), and ED nurses (2/37, 5%; Table 1). The baseline characteristics of health care professionals who solely filled out the primary surveys did not vary statistically significantly in age (*P*=.98) or sex (*P*=.28) as compared to those who filled out both surveys. A total of 15 health care professionals, 5 per hospital, participated in the web-based, semistructured interviews, of which 60% (9/15) were female.

**Table 1:** Baseline characteristics of health care professionals included in a mixed methods evaluation of Direct Discharge.

Characteristics		Survey (n=37)		Interview (n=15)
Female, n (%)		23 (62)		9 (60)
Age (years), median (IQR)		38 (32-45)		40 (32-44)
**Hospital,** **n** **(%)**				
	Hospital A	19 (51)	5 (33)	
	Hospital B	11 (30)	5 (33)	
	Hospital C	7 (19)	5 (33)	
**Current function,** **n** **(%)**				
	Trauma surgeon	5 (13)	3 (20)	
	Orthopedic surgeon	1 (3)	0 (0)	
	Emergency physician	8 (22)	2 (13)	
	Surgery resident in training	8 (22)	3 (20)	
	Surgery resident not in training	6 (16)	1 (7)	
	Plaster technician	7 (19)	3 (20)	
	ED^a^ nurse	2 (5)	3 (20)	
	Total	37 (100)	16 (100)	

^a^ED: emergency department.

### Implementation

Qualitative data showed that the implementation strategy varied between hospitals and was adjusted to improve the local fit (eg, available hours of the helpline or brace brands). Changes in tasks, immobilization material, and the number of follow-up appointments influenced the implementation experience the most. Some nurses reported that coworkers had difficulties applying the braces, sometimes because their schooling during implementation was suboptimal or delayed, for example, because someone was ill (quote 1 in Table 2). Health care professionals reported that adequate schooling and involvement of the previous responsible health care professional in the implementation process were essential to executing the DD protocol adequately. Mainly because the ED nurse, now responsible for applying the braces, had less experience immobilizing fractures than the plaster technician.

**Table 2:** Health care professionals’ quotes and associated feasibility parameters.

Theme and quote number		Quote	Health care professional
**Implementation of Direct Discharge among health care professionals**			
	1	Some of my colleagues have difficulty with the materials. How does it work and what goes where? It takes a bit longer for some of them to get the hang of it. Having to learn so many new things sometimes causes resistance.	ED^a^ nurse
**Acceptability of the Direct Discharge among health care professionals**			
	2	This is a significant improvement for patients and appeals to their autonomy and control, as well as their own influence on the healing process. I believe it is motivating and in line with the current times.	ED nurse
	3	I no longer have to do these routine outpatient clinical check-ups. I could only provide limited contributions besides providing information, allowing me to have more peace and tranquility in the clinic. I can use that time for other patients to add more value.	Trauma surgeon
	4	Both among doctors and nurses, DD has been widely embraced and well implemented, but for both professions, it requires valuable extra minutes due to additional explanation. Currently, the workload is very high.	Resident
	5	But if that care is taken away from us, I do believe that we have a responsibility towards the patients to ensure the proper transition of that care.	Plaster technician
**Preliminary efficacy of the Direct Discharge among health care professionals**			
	6	We now have a tool in our hands to change healthcare without it deteriorating, which convinces people who tended towards over-treatment.	ED physician
	7	The quality is not affected, assuming the doctor was already good. It is mainly more efficient. Information provision has improved. It has become more modern. I think DD is not worse, but we’re not certain yet.	Trauma surgeon
	8	We are sometimes called about 2-3 times per day on the fracture line. I don’t think that’s a bad score.	Plaster technician
**Demand for Direct Discharge among health care professionals**			
	9	I was already familiar with DD because I was looking for good and reliable information on injuries during my training as an ED nurse. I came across the article published by OLVG and started using the app in my work.	ED nurse
	10	Every day, a few patients are treated through the app. I am starting to notice the reduction in daily practice!	Trauma surgeon
	11	In my work, it has changed that we see less patients, but new things have also been added. However, we no longer see minor injuries. The easier type of care has decreased a bit.	Plaster technician
**Applicability of the Direct Discharge among health care professionals**			
	12	I see the advantage of a brace instead of a cast, a great improvement. I would also prefer DD myself.	Resident
	13	The walking boot is difficult to fit, which poses a risk of misuse. This results in most complications being caused by misuse. What does that do to the recovery process?	ED physician

^a^ED: emergency department.

### Acceptability

#### Overview

Quantitative data showed a median satisfaction with treatment of 7.8 (IQR 6.8-8.9) on a 10-point scale. This finding complemented qualitative data, as most health care professionals were satisfied with DD (quote 2 in Table 2). Health care professionals suggested that in-app patient feedback, such as pain scores or patient-reported outcome measures, could further increase health care professional satisfaction levels and address health care professionals’ possible concerns about adequate recovery. Some health care professionals were hesitant about DD due to its novelty, limited education during implementation, changes in work activities, and concerns about the short- and long-term outcomes of certain injuries.

#### VFC App Acceptability

The introduction of the VFC app at the ED yielded both advantages and disadvantages. Both qualitative and quantitative data showed that introducing the VFC app and treatment information in the ED required more time than the traditional treatment (19/37, 52%; Multimedia Appendix 5). Qualitative data showed that this time decreased once DD was fully implemented but remained longer than traditional treatment. Despite the increased time to inform patients, the perceived reduction in logistics at the outpatient clinic was perceived as more valuable (quotes 3 and 4 in Table 2). Both data sources underline the benefits of the app in terms of uniform, on-demand, and adequate information for patients after discharge. The downsides of the VFC app include less personal health care and the current limited language availability (Dutch and English).

#### Workload and Job Satisfaction

Qualitative data showed that workload and job satisfaction decreased slightly after the introduction of DD. Quantitative data did not support this, as no statistically significant differences between expectations and experiences were found regarding workload (*P*=.37) and increased job satisfaction (*P*=*.*42). Plaster technicians reported that the introduction of DD has led to losing a “fun” part of their job. Some reported they could provide less service for patients and felt responsible for educating ED nurses who had less experience with the immobilization of fractures compared with them (quote 5 in Table 2).

### Preliminary Efficacy

#### Quality of Care

Quantitative data showed that the quality of care with the DD protocol is perceived as comparable to traditional treatment (25/37, 67%). Health care professionals reported an improvement in the quality of information and uniformity (30/37, 82%; Multimedia Appendix 6). No statistically significant differences were found between expectations and experiences of quality of care (*P*=.86), quality of information (*P*=.42), and quantity of information (*P*=.18). Qualitative data supported these findings, highlighting the benefits of uniform, injury-specific information (quotes 6 and 7 in Table 2). In both the survey and interviews, most (27/37, 73%) health care professionals reported a reduction in outpatient follow-up and injury-related imaging. Qualitative data showed that (orthopedic) trauma surgeons experienced the reduction as beneficial, whereas some plaster technicians experienced the reduction as a disadvantage. Most residents and ED physicians reported that the logistical benefits at the outpatient clinic outweighed the slight increase in time at the ED.

#### Perceived Safety

Both data sources showed that most (29/37, 79%) health care professionals perceived DD as safe and that sufficient scientific evidence exists to treat patients safely. Health care professionals assumed that patients had fully recovered if they did not contact the hospital again. Nevertheless, they proposed a numerical feedback system in the VFC app to ensure adequate recovery and alleviate concerns about the poor long-term functional outcomes of their patients. The frequency of helpline use was low (≤5 times per week; quote 8 in Table 2). Some residents reported that the introduction of DD decreased their exposure to low-complex traumatic injuries, which might influence their learning curve in the future. Furthermore, the lack of follow-up introduced the tendency for some residents to be more explanatory at the ED. However, some residents stated that during crowding at the ED, they limited their explanation to downloading the VFC app with minimal instructions. The frequency of calling varied per injury, with patients with a greenstick fracture rarely requiring contact. Reasons to call the helpline were similar among the 3 centers. Patient questions were related to a poor recall of the ED visit, suboptimal information provision at the ED, doubts about their recovery, or insufficient reading of the app’s content. The daily multidisciplinary radiologic evaluation and helpline were considered effective safety nets.

#### Demand

Qualitative analysis revealed that some health care professionals had previous knowledge or experience with the DD protocol (quote 9 in Table 2). The COVID-19 pandemic positively influenced their perceptions of digitally assisted care. Some health care professionals reported that it fits the general demand to develop more efficient outpatient follow-up models and that the DD protocol is an example of “tomorrow’s health care.” Reduced outpatient follow-up, hospital use (eg, treatment rooms or parking lots), and staff were mentioned as benefits. Health care professionals expected the DD protocol to stay and intended to continue using it. Health care professionals reported that the DD protocol could improve patient empowerment and enhance self-management and independence, especially among younger patients. Health care professionals had different experiences integrating the DD protocol into their daily activities. Orthopedic trauma surgeons perceived a decrease in patients at the outpatient clinic following DD implementation and stated that their workload was starting to decrease due to DD (quote 10 in Table 2). Plaster technicians also reported a decrease in patients. However, they perceived this sometimes as a disadvantage as they no longer treated these low-complex injuries, which is considered a loss of a fun part of their job. No reduction in workload was perceived among plaster technicians due to additional tasks and an increase in a patient population with more complex injuries (quote 11 in Table 2).

Residents reported that extensive information has changed from the ED and outpatient clinic to solely the ED. The ED nurses reported applying fewer casts and more orthoses, such as braces or walking boots.

#### Applicability

Before implementation, some health care professionals expressed concerns about the incorrect use of immobilization materials. However, after implementation, the types of immobilization materials were perceived as adequate, with 74% (27/37) of health care professionals finding the braces easy to use for patients (Multimedia Appendix 7). The braces were less immobilizing than a cast, which was perceived as a benefit (quote 12 in Table 2). The less immobilizing treatment regimens could result in an earlier return to full function. However, some were concerned that the braces could lead to inferior long-term functional outcomes, even though the scientific evidence for these concerns was lacking (quote 13 in Table 2).

## Discussion

### Principal Findings

Dutch health care professionals considered the DD protocol a safe and satisfactory alternative to traditional treatment, leading to a perceived decrease in SHU. Although providing information with DD required more (explanation) time for residents and physicians at the ED, the logistical benefits (ie, reduced number of follow-up appointments) outweighed the slight increase in time. Before and after implementation, no statistically significant differences were found regarding workload or job satisfaction. However, qualitative data reported benefits in workload and job satisfaction for trauma surgeons and residents, and disadvantages for some plaster technicians in terms of job satisfaction and workload. Furthermore, health care professionals reported increased quality of information and comparable quality of care. Almost all health care professionals would like to continue using DD after implementation. To improve DD, several new app functionalities were suggested, and the earlier involvement of stakeholders who performed new tasks was suggested.

### Comparison With Existing Literature

The study results show that DD is an acceptable alternative to traditional follow-up for health care professionals. Health care professionals reported similar, high satisfaction scores with treatment as previously reported patient satisfaction scores for similar protocols [18,33]. To further improve health care professionals’ satisfaction scores, health care professionals in this study suggested a short numerical feedback system to monitor injury recovery remotely (eg, recovery questions after 3 months). In the literature, health care professionals have also mentioned this as an important feature of eHealth developed for patients with musculoskeletal injuries [34,35]. A challenge to incorporating this is that these functions would require a more enhanced app that complies with current laws and regulations for data storage and requires substantial financial investment. In addition, this request might also be a sign of early-stage adoption, where health care professionals feel a bit uncertain about the patients they would normally see for follow-up but who are now out of sight. As time progresses, it seems likely they will feel more comfortable not seeing these patients anymore, as this is then considered standard of care.

Preliminary efficacy was partly in line with the literature [18,20,33]. Most health care professionals considered DD a safe alternative to face-to-face follow-up, leading to a perceived reduction of SHU. This finding is consistent with previous articles, which concluded that DD is safe based on low, comparable complication numbers, with significantly reduced SHU compared to traditional treatment [9,18,33]. The perceived reduction in SHU varied among stakeholders. The reduction was perceived as most beneficial in qualitative data regarding workload for (orthopedic) trauma surgeons and least beneficial for plaster technicians, as they enjoyed this particular part of their workload. This study has been unable to demonstrate statistically significant differences regarding workload and job satisfaction before and after implementation. Even though the introduction of DD at the ED increased valuable treatment time for some stakeholders, which decreased but remained longer after implementation, almost all reported that the benefits of less SHU outweighed this downside. These findings are interesting, as the increase in time has been reported as one of the most important personal barriers to implementing and adopting eHealth but was not reported as an important disadvantage in this study [14,35].

Similar quality of care and increased quality of information after the introduction of DD are in accordance with earlier findings [11,20,36]. Particular advantages of DD were uniform, injury-specific, and on-demand information, consistent with a study pointing out the current heterogeneity in treatments for these injuries and the demand for uniform treatment [37]. The disadvantages of DD were the care being less personal, the loss of care activities for several stakeholders, and the inability to monitor recovery. A previous study described that VFCs might influence learning curves as residents become less experienced with the follow-up and full recovery of these injuries [38]. This was not supported by our results. However, a lack of follow-up has led to a tendency for some (often inexperienced) residents to be more explanatory at the ED to ensure the patient had received all information during the only contact moment. To reassure themselves and identify the small group of patients with persistent complaints, some health care professionals suggested a feedback system to ensure they had recovered adequately. Such a system should not increase workload but only filter patients with remaining questions, pain, or complications. This should be developed with patients and health care professionals and could contain anchor-based questions or patient-reported outcome measures with predefined cut-off values.

Health care professionals report that DD fits the public demand to develop more efficient (outpatient) care, and digital assistance could help deal with constrained resources. This partly aligns with the literature, as attitudes toward the usefulness of eHealth vary [39-41]. Almost all health care professionals would like to continue using DD after its implementation. This finding complements current literature showing the widespread implementation of DD protocols and VFCs in the United Kingdom, Australia, New Zealand, and India [8,33,42,43]. This further strengthens the idea of at least adequate acceptance and the general applicability of this concept in different countries. The applicability of DD varied among stakeholders and was influenced by the increase or loss of tasks after implementation of the protocol and their experience with that particular increase or loss of tasks. Even though evaluation of applicability for health care professionals and health care professional satisfaction with the protocol seems vital to determining feasibility, it has not been reported previously for DD protocols, despite the many centers that have implemented similar protocols. Health care professionals reported that early stakeholder involvement during implementation could be beneficial to cope with the changes in tasks and transfer of knowledge, which aligned with the results of Logishetty [21] reporting the importance of early stakeholder involvement during VFC implementation in a quality improvement approach. The lack of early involvement of stakeholders and limited feasibility among health care professionals are known risks for nonadoption or abandonment [3,44,45].

### Strengths and Limitations

This study has several strengths. To date, this is the first study to explore the in-depth views of several stakeholders involved in DD protocols and VFCs. The COVID-19 pandemic has accelerated the implementation of DD in the Netherlands, emphasizing the demand for a shift in patient care, where eHealth alternatives have become the new standard [46]. The views and perceptions of health care professionals about different eHealth services are valuable to further tailor these services to their needs and preferences. A second strength of this study is the multidisciplinary involvement of the research team during evaluation and analysis. This approach ensured that the data were analyzed from all possible angles. A third strength is a mixed methods approach to evaluating DD because the separate collection of both data sources combined with the triangulation increased the likelihood of our results being a realistic representation of the daily clinical activities of the involved health care professionals. The fourth strength is the use of a validated framework to evaluate DD, which allowed for a structured insight into each feasibility parameter. The fifth strength was the heterogeneity in the sizes and locations of the three study sites. This allowed us to investigate the applicability within different types of hospitals.

This study also has limitations that need to be addressed. The first limitation is the small quantitative sample size and low response rate. Even though the response rate among health care professionals is 10% to 15% lower compared to patient studies, the quantitative sample size remains limited [47]. Nevertheless, for almost all parameters, quantitative and qualitative data were similar, indicating a realistic response from most health care professionals. A third limitation is that ED nurses were not involved in the initial design of this study and did not provide quantitative data. Nevertheless, they have been included in the qualitative data to strengthen the study results.

### Implications for Clinicians and Policy Makers and Future Perspectives

DD protocols reduce SHU without negatively influencing PHU, satisfaction, complications, or functional outcomes [17]. By assisting patients through the VFC app, they can receive care at home, potentially reducing health care costs [9]. Health care costs in the Netherlands have increased drastically, particularly due to specialized in-hospital care. In addition, there is a growing shortage of personnel. Digitally assisted solutions are suggested to cope with the rise of these costs and limited personnel, but implementation remains difficult [14]. The technical (eg, compliance and complication numbers) and logistic outcomes of this concept are well explored, but the social and cultural elements of DDs have not been explored. These findings help optimize future implementation strategies for eHealth in orthopedic and trauma surgery by providing preconditions and learning lessons such as early stakeholder involvement. These findings might be generalizable for other short treatment processes in other (surgical) departments with high volumes of relatively low-complex surgical patients (eg, low-complex dermatology or otorhinolaryngology).

As DD is introduced rapidly to cope with constrained financial and human resources and health care professionals expect DD to stay, a thorough evaluation of caregivers and patients is essential to ensuring sustainable adoption. Results of this study have improved our DD protocol and have led to 2 corresponding major points of improvement among all stakeholders and many points of improvement, such as ideas for illustrations, adjusted language (levels), and adjustable font size from 1 or more health care professionals. Future studies should focus on co-designing numerical feedback with patients and health care professionals. Furthermore, a thorough evaluation of patient perspectives should be performed to gain insight into the end users’ perspective on this innovation. Particularly to investigate the potential link between different levels of literacy (health literacy, digital literacy, and literacy) and health equity, as suggested in previous studies [48,49].

### Conclusion

Health care professionals perceive DD as an acceptable, applicable, safe, and efficacious alternative to traditional treatment. A short numerical feedback system could alleviate concerns about a full recovery and further improve DD protocols. DD can reduce SHU, which is important in times of constrained resources. Nonetheless, both advantages and disadvantages should be considered while evaluating this type of protocol. In the future, clinicians and policy makers can use these insights to further optimize and implement DD and VFC in clinical practice and guidelines.

## Appendix

Multimedia Appendix 1 Assessment of Good Reporting on a Mixed Methods Study (GRAMMS) criteria for the study.

Multimedia Appendix 2 Additional criteria and immobilization for low-complex, traumatic orthopedic injuries included in the Direct Discharge Protocol.

Multimedia Appendix 3 Surveys used to evaluate Direct Discharge among health care professionals.

Multimedia Appendix 4 Topic list for health care professionals the Direct Discharge protocol.

Multimedia Appendix 5 5-Point Likert scale regarding applicability of the Direct Discharge among health care professionals.

Multimedia Appendix 6 5-Point Likert scale regarding preliminary efficacy of the Direct Discharge among health care professionals.

Multimedia Appendix 7 5-Point Likert scale regarding applicability of the Direct Discharge among health care professionals.

## Abbreviations

DD: Direct Discharge
ED: emergency department
GRAMMS: Good Reporting on a Mixed Methods Study
OLVG: Onze Lieve Vrouwe Gasthuis
PHU: primary health care use
REDCap: Research Electronic Data Capture
SHU: secondary health care use
VFC: Virtual Fracture Clinic
