# Supplementary material for: Health Care Professionals’ Experiences With a Mobile Self-Care Solution for Low Complex Orthopedic Injuries: Mixed Methods Study
Source: JMIR Mhealth Uhealth. 2024 Feb 2;12:e51510. doi: 10.2196/51510 (PMC10873799; doi:10.2196/51510)
Supplement: Multimedia Appendix 1 [file mhealth_v12i1e51510_app1.docx]

**Multimedia Appendix 1.** Assessment of GRAMMS criteria for the study: ‘*Healthcare professionals experiences with an mHealth self-care solution for low complex orthopaedic Injuries: a mixed method study’*

|  |  | Yes | Yes, but | no | NEI or N/A |
| --- | --- | --- | --- | --- | --- |
| 1. | Is the quantitative component feasible? | X |  |  |  |
| 2. | Is the qualitative component feasible? | X |  |  |  |
| 3. | Is the mixed methods design feasible? | X |  |  |  |
| 4. | Have both qualitative and quantitative components been completed? | X |  |  |  |
| 5. | Were some quantitative methods planned but not executed? |  | X, the sample size was too small to perform subgroup analysis |  |  |
| 6. | Were some qualitative methods planned but not executed? |  |  | X |  |
| 7. | Did the mixed methods design work in practice | X |  |  |  |

**Table S1.** Assessment of the success of the mixed methods study

NEI, not enough information

N/A, not applicable

**Table S2.** Assessment of the mixed methods design of the study

|  |  | | Yes | Yes, but | no | NEI or N/A |
| --- | --- | --- | --- | --- | --- | --- |
| 1. | Is the use of mixed methods research justified? | | X |  |  |  |
| 2. | Is the design for mixing methods described? | |  |  |  |  |
|  |  | Priority | X |  |  |  |
|  |  | Purpose | X |  |  |  |
|  |  | Sequencing | X |  |  |  |
|  |  | Stage of integration |  |  |  |  |
| 3. | Is the design clearly communicated? | | X |  |  |  |
| 4. | Is the design appropriate for addressing the research questions? | | X |  |  |  |
| 5. | Has rigour of the design been considered (proposal) or adhered to (report)? | | X |  |  |  |

NEI, not enough information

N/A, not applicable

**Table S3.** Assessment of the quantitative component of the mixed method study

|  |  | Yes | Yes, but | no | NEI or N/A |
| --- | --- | --- | --- | --- | --- |
| 1. | Is the role of each method clear? | X |  |  |  |
| 2. | Is each method described in sufficient detail? | X |  |  |  |
| 3. | Is each method appropriate for addressing the research question? | X |  |  |  |
| 4. | Is the approach to sampling and analysis appropriate to this purpose | X |  |  |  |
| 5. | Is there expertise among applicants/authors? | X |  |  |  |
| 6. | Is there expertise on the team to undertake each method? | X |  |  |  |
| 7. | Have issues of validity been addressed for each method? | X |  |  |  |
| 8 | Has the rigour of any method been compromised |  | Yes, but this has clearly been described |  |  |
| 9. | Is each method sufficiently developed for each purpose? | X |  |  |  |
| 10. | Is the (intend) analysis sufficiently sophisticated | X |  |  |  |

NEI, not enough information

N/A, not applicable

**Table S4.** Assessment of the qualitative component of a mixed method study

|  |  | Yes | Yes, but | no | NEI or N/A |
| --- | --- | --- | --- | --- | --- |
| 1. | Is the role of each method clear? | X |  |  |  |
| 2. | Is each method described in sufficient detail? | X |  |  |  |
| 3. | Is each method appropriate for addressing the research question? | X |  |  |  |
| 4. | Is the approach to sampling and analysis appropriate to this purpose |  | X, the interviews have been summarized |  |  |
| 5. | Is there expertise among applicants/authors? | X |  |  |  |
| 6. | Is there expertise on the team to undertake each method? | X |  |  |  |
| 7. | Have issues of validity been addressed for each method? | X |  |  |  |
| 8 | Has the rigour of any method been compromised |  | This has been clearly described in limitations |  |  |
| 9. | Is each method sufficiently developed for each purpose? | X |  |  |  |
| 10. | Is the (intend) analysis sufficiently sophisticated | X |  |  |  |

NEI, not enough information

N/A, not applicable

**Table S5.** Assessment of integration in the mixed method study

|  |  | Yes | Yes, but | no | NEI or N/A |
| --- | --- | --- | --- | --- | --- |
| 1. | Is the type of integration stated? | X |  |  |  |
| 2. | Is the type of integration appropriate for the design? | X |  |  |  |
| 3. | Has enough time been allocated for integration? | X |  |  |  |
| 4. | IS the approach to integration detailed in terms of working together as a team? | X |  |  |  |
| 5. | Does the dissemination strategy detail how the mixed methods will be reported in final reports and peer-reviewed publications? | X |  |  |  |
| 6. | Are the personnel who participate in the integration clearly identified? | X |  |  |  |
| 7. | Did appropriate members of the team participate in the integration? | X |  |  |  |
| 8 | Is there evidence of communication within the team? | X |  |  |  |
| 9. | Has rigour been compromised by the process of integration |  |  | X |  |

NEI, not enough information

N/A, not applicable

**Table S6.** Assessment of the interferences made in completed reports of the mixed method study

|  |  | Yes | Yes, but | no | NEI or N/A |
| --- | --- | --- | --- | --- | --- |
| 1. | Is there clarity about which results have emerged from which methods? | X |  |  |  |
| 2. | Are inferences appropriate? | X |  |  |  |
| 3. | Are the results of all the methods considered sufficiently in the interpretation? | X |  |  |  |

NEI, not enough information

N/A, not applicable

**Textbox S1**: Good Reporting of A Mixed Methods Study (GRAMMS)

(1 ) Describe the justification for using a mixed methods approach to the research question

(2) Describe the design in terms of the purpose, priority and sequence of methods

(3) Describe each method in terms of sampling, data collection and analysis

(4) Describe where integration has occurred, how it has occurred and who has participated in it

(5) Describe any limitation of one method associated with the present of the other method

(6) Describe any insights gained from mixing or integrating methods
