# Supplementary material for: Health Care Professionals’ Experiences With a Mobile Self-Care Solution for Low Complex Orthopedic Injuries: Mixed Methods Study
Source: JMIR Mhealth Uhealth. 2024 Feb 2;12:e51510. doi: 10.2196/51510 (PMC10873799; doi:10.2196/51510)
Supplement: Multimedia Appendix 3 [file mhealth_v12i1e51510_app3.docx]

**Multimedia Appendix 3.** Surveys used to evaluate Direct Discharge among healthcare professionals

***Study ID 1 (Event 1)***

**Personal Information** *Page 1*

Dear colleague,

In 2021, Direct Discharge, a new standard treatment, is being introduced for patients with simple and stable injuries. With this treatment, patients no longer receive routine outpatient follow-up and are treated with a brace instead of a cast. They receive extensive explanation at the Emergency Department, which is summarized in a smartphone application. The smartphone application consists of a digital brochure and videos with exercises. During the introduction of Direct Discharge, the Virtual Fracture Care Research Group will conduct a feasibility study.

In this study, we aim to determine to what extent Direct Discharge in its current form fits into Dutch emergency care and outpatient orthopedic and traumatology care, and how it is received by both patients and professionals. In addition, we will also determine whether, how, and where Direct Discharge promotes the efficiency of care, while maintaining the quality and satisfaction of patients and healthcare professionals. This questionnaire is part of this feasibility study, in which we will map out the work situation of caregivers involved in low-complexity injuries after the introduction of Direct Discharge. Filling out the following questionnaire takes about eight minutes.

Three months after the implementation of Direct Discharge, you will receive another request to fill out a questionnaire. Thank you in advance for your cooperation.

Best regards,

Jelle Spierings

MD, PhD-candidate

| **Baseline data** |
| --- |

I filled out this survey on:

(DD-MM-YYYY)

| 19-04-2023 11:58 [projectredcap.org](https://projectredcap.org) | 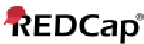 |
| --- | --- |

***Study ID 1 (Event 1)***

*Page 2*

1. My current occupation is

- Trauma surgeon
- Orthopedic surgeon
- Surgeon
- ED physician
- ED resident.
- Physician Assistant ED
- Orthopedic Surgery resident
- General surgery resident
- Other
- Plaster technician

2.) I currently work in the ….

- Spaarne Gasthuis
- Gelre Ziekenhuis
- Slingeland Ziekenhuis

3.) Year of birth [YYYY]

4.) Sex Male

Female

| 19-04-2023 11:58 [projectredcap.org](https://projectredcap.org) | 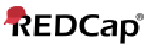 |
| --- | --- |

***Study ID 1 (Event 1)***

**Expectations** *Page 3*

**Survey Expectations**

19-04-2023 11:58 [projectredcap.org](https://projectredcap.org)


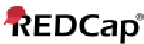


|  | Totally disagree | Disagree | Neutral | Agree | Totally agree |
| --- | --- | --- | --- | --- | --- |
| 5.) I expect that with the introduction of DD, there will be a decrease in outpatient follow-up. |  |  |  |  |  |
| 6.) I expect that with the introduction of DD, there will be a decrease in attributive radiological imaging |  |  |  |  |  |
| 7.) I expect that with the introduction of DD, there will be a decrease in attributive radiological imaging |  |  |  |  |  |
| 8.) I expect that Direct Discharge will result in better information provision for patients. |  |  |  |  |  |
| 9.) I expect that with the introduction of Direct Discharge, patients with a simple and stable injury can be adequately treated without further physical consultation at the orthopedic or surgical outpatient clinic. |  |  |  |  |  |
| 10.) I expect that with the introduction of Direct Discharge, patients with a simple and stable injury can be adequately treated using the self-care application: 'the Virtual Fracture Care App.' |  |  |  |  |  |
| 11.) I expect that with the introduction of DD, the workload will decrease for treating (para)medical professionals in the Emergency Department. |  |  |  |  |  |
| 12.) I expect that with the introduction of DD, the workload for doctors and medical specialists at the Surgical or Orthopedic outpatient clinic will decrease. |  |  |  |  |  |
| 13.) I expect that the introduction of DD will not lead to an increase in workload for general practitioners or physiotherapists in the catchment area of my hospital. |  |  |  |  |  |
| 14.) I expect that DD will align well with my daily work activities. |  |  |  |  |  |
| 15.) I expect that DD is safe for patients in my hospital. |  |  |  |  |  |
| 16.) I expect that the quality of care for low complexity fracture care will remain the same after the introduction of Direct Discharge. |  |  |  |  |  |
| 17.) I expect that my job satisfaction will increase after the introduction of Direct Discharge. |  |  |  |  |  |
| 18.) I expect that Direct Discharge has a positive effect on my workload. |  |  |  |  |  |
| 19.) I expect that the quality of care for low complexity fractures will remain the same after the introduction of Direct Discharge. |  |  |  |  |  |
| 20.) I expect that the orthoses will be easily applicable for patients. |  |  |  |  |  |
| 21.) I expect that the quality of information will improve for patients with Direct Discharge |  |  |  |  |  |
| 22.) I expect that the quantity of information  for patients with Direct Discharge in the  Emergency Department is … . | Way too little | Not enough | Just right | Too much | Way too much |

***Study ID 1 (Event 1)***

**Expectations – Residents & plaster technicians** *Page 12*

**Expectations – Residents**

19-04-2023 11:58 [projectredcap.org](https://projectredcap.org)


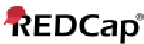


- Very low
- Low
- Neutral
- High
- Very high
- Totally disagree
- Disagree
- Neutral
- Agree
- Fully agree

25.) After the introduction of DD I expect my job satisfation will

Increase.

24.) What is your perceived workload at the ED regarding simple and stable injuries?

- Very low
- Low
- Neutral
- High
- Too high

23.) What is your opinion regarding workload during plaster room supervision?

***Study ID 1 (Event 1)***

*Page 13*

on my workload.


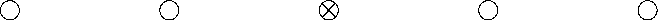


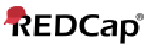


**Perceived workload at the plaster room**

**Perceived workload regarding simple and stable injuries at the ED**

26.) Further remarks?

***Study ID 1 (Event 1)***

**End of first Survey** *Page 15*

The results of these questions will be used to ask about your expectations. In three months, we would like to approach you again to ask how you have experienced the change in the healthcare process. Based on both questionnaires, short interviews will be scheduled with patients, plaster cast technicians, nurses, and doctors.

27.) After these surveys we would like to contact you to schedule a

- Yes
- No

Semi-structured interview after consent.

Do you give consent to approach you?

| **In case you do not give consent to contact you for a semi-structured interview we will send you the second questionnaire in 3 months** |
| --- |

28.) What is your e-mailadress? [EMAIL]

| 19-04-2023 11:58 [projectredcap.org](https://projectredcap.org) | 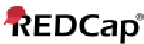 |
| --- | --- |

***Study ID 1 (T1 Medical Professionals)***

**Experiences survey** *Page 16*

The following (short) questionnaire explores your experiences with DD and takes around 5 minutes to fill out.

**The next questions are about acceptation of DD**

33.) Do you have any further remarks?

**Demand**

19-04-2023 11:58 [projectredcap.org](https://projectredcap.org)


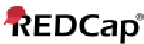


|  | Totally disagree | Disagree | Neutral | Agree | Totally agree |
| --- | --- | --- | --- | --- | --- |
| 29.) The introduction of DD leads to a decrease in outpatient follow-up. |  |  |  |  |  |
| 30.) The introduction of DD leads to a decrease in attributive radiological imaging |  |  |  |  |  |
| 31.) The introduction of DD leads to a decrease in treatments times |  |  |  |  |  |
| 32.) Patients with simple and stable injuries are now treated with DD instead of the outpatient clinic |  |  |  |  |  |

|  | Totally disagree | Disagree | Neutral | Agree | Totally agree |
| --- | --- | --- | --- | --- | --- |
| 34.) The introduction of DD has decreased my workload |  |  |  |  |  |
| 35.) DD has increased my job satisfaction |  |  |  |  |  |
| 36.) DD improves the patient information for patient with simple and stable injuries |  |  |  |  |  |
| 37.) The quantity of information is […] for our patients | Too little | limited | Just right | Too much | Way too much |

38.) How satisfied are you with DD?

(0 = very unlikely en 100 = very likely) 0 50 100

*(Place a mark on the scale above)*

Do you have any further remarks?

**Applicability**

|  | Totally disagree | Disagree | Neutral | Agree | Totally agree |
| --- | --- | --- | --- | --- | --- |
| 39.) DD fits seamlessly in my daily activities |  |  |  |  |  |
| 40.) DD is safe for our patients |  |  |  |  |  |
| 41.) The quality of care has stayed similar after the introduction of DD |  |  |  |  |  |
| 42.) The orthoses are easy to apply |  |  |  |  |  |
| 43.) I would like to continue using DD. |  |  |  |  |  |

44.) Do you have any further remarks to improve the protocol?

**Thank you for your participation!**
