# Supplementary material for: Health Care Professionals’ Experiences With a Mobile Self-Care Solution for Low Complex Orthopedic Injuries: Mixed Methods Study
Source: JMIR Mhealth Uhealth. 2024 Feb 2;12:e51510. doi: 10.2196/51510 (PMC10873799; doi:10.2196/51510)
Supplement: Multimedia Appendix 4 [file mhealth_v12i1e51510_app4.docx]

**Multimedia Appendix 4.** topic list for healthcare professionals the Direct Discharge protocol

**Topic list used for healthcare professionals to evaluate the Direct Discharge Protocol**

**Acceptation**

- *Satisfaction, perceived fit (appropriateness) and perceived quality of care*
- *Year of birth + years of experience*
- How would you describe your own involvement with Direct Discharge?
- What is good about Direct Discharge that should not change? Anything else? And one more thing?
- What could be improved about Direct Discharge? Anything else? And one more thing?
- What expectations did you have of Direct Discharge beforehand? Can you explain that?
- How satisfied are you with Direct Discharge? Can you explain that? And if you were to give a rating (0-10), what would you choose? What factors contribute to your choice?

**Demand**

- *Interest and intention to continue use*
- To what extent does Direct Discharge align with your job responsibilities?
- What are the benefits of Direct Discharge for your work? And the drawbacks?
- To what extent does Direct Discharge meet a need? Can you explain that? Is it always the case? And after COVID?
- If the choice is up to you, would you continue to use Direct Discharge? Can you explain that?

**Implementation**

- *Degree of performance of tasks, usability, factors of influence of DD and VFC-app*
- *Barriers and facilitators that simplify or complicate the implementation*.
- ***What has changed since your hospital started using Direct Discharge? (initial question)***
- How has that been for you? And for your daily tasks? Can you provide an example? (followed by Demand)
- What are factors/things that make Direct Discharge and the use of the VFC-app easy? And what else? (If you have to prioritize them, which one would be at the top? And at 2nd place? 3rd place?)
- What are factors/things that make Direct Discharge and the use of the App difficult or obstructive? And what else? (If you have to prioritize them, which one would be at the top? And at 2nd place? 3rd place?)

**Applicability**

- *Expected ad- and disadvantages*
- *Integration of th DD-protocol in daily routine*
- *Perceived fit in local infrastructure of care at patient-, organization- and healthcare professional level*
- *Perceived durability of protocol at patient-, organization- and healthcare professional level*
- What benefits do you see in using Direct Discharge? And for patients? And for the organization? And for the professionals? What is the biggest advantage?
- What drawbacks do you see in using Direct Discharge? And for patients? And for the organization? And for the professionals? What is the biggest disadvantage?
- To what extent do you expect to continue working with Direct Discharge next year? Can you explain that?

*If relevant to the conversation:*

- How well does Direct Discharge fit with patients on a scale of 0-10? What factors contribute to your rating?
- How well does Direct Discharge fit with you as a professional on a scale of 0-10? What factors contribute to your rating? How could it be improved?

**Preliminary-efficacy.**

- *Perceived quality of care and perceived safety*
- *Experience of medical professionals*
- To what extent do you believe that Direct Discharge is safe for patients? Can you explain that? Are there any exceptions? What leads you to say this?
- To what extent has the introduction of Direct Discharge affected care in the emergency department? Can you provide an example?
- What has Direct Discharge brought about? What effects do you see in practice? And for patients? And for professionals?
- To what extent does Direct Discharge affect the quality of care? Can you explain that?
- To what extent do you agree with the statement: "Direct Discharge is the way to make care for our patients efficient and future-proof"? Can you explain that?
