# Supplementary material for: Health Care Professionals’ Experiences With a Mobile Self-Care Solution for Low Complex Orthopedic Injuries: Mixed Methods Study
Source: JMIR Mhealth Uhealth. 2024 Feb 2;12:e51510. doi: 10.2196/51510 (PMC10873799; doi:10.2196/51510)
Supplement: Multimedia Appendix 7 [file mhealth_v12i1e51510_app7.docx]

**Multimedia Appendix 7**. 5-Point Likert scale regarding applicability of the Direct Discharge among health care professionals.

**
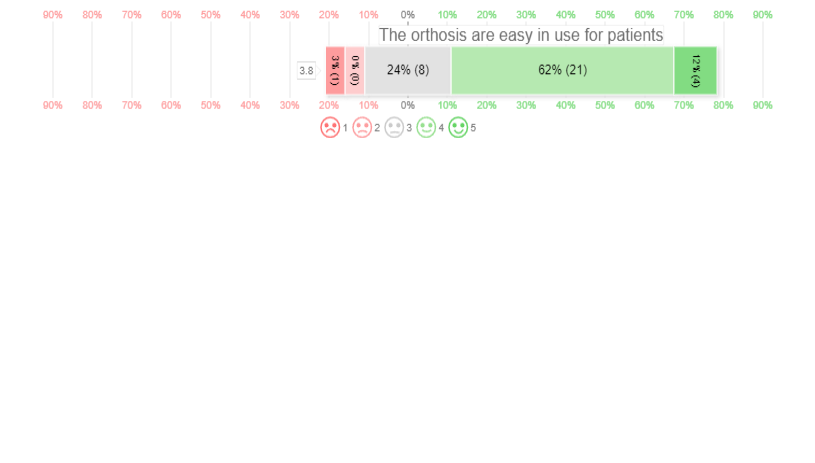
**

󠇣 = mean

5-point Likert Scale with Red or 1 indicating totally disagree and green or 5 indicating totally agree
